# Circular RNA hsa_circ_0000700 promotes cell proliferation and migration in Esophageal Squamous Cell Carcinoma by sponging miR-1229

**DOI:** 10.7150/jca.47112

**Published:** 2021-03-05

**Authors:** Jun Fang, Wen Hao Ji, Fang Zheng Wang, Tie Ming Xie, Lei Wang, Zhen Fu Fu, Zhun Wang, Feng Qin Yan, Qi Liang Shen, Zhi Min Ye

**Affiliations:** 1 Department of Radiotherapy, The Cancer Hospital of the University of Chinese Academy of Sciences (Zhejiang Cancer Hospital), Institute of Basic Medicine and Cancer (IBMC), Chinese Academy of Sciences, Hangzhou, Zhejiang, 310022, China.; 2 Key Laboratory of Head & Neck Cancer Translational Research of Zhejiang Province, Hangzhou, Zhejiang, 310022, China.; 3 Department of Radiology, The Cancer Hospital of the University of Chinese Academy of Sciences (Zhejiang Cancer Hospital), Institute of Basic Medicine and Cancer (IBMC), Chinese Academy of Sciences, Hangzhou, Zhejiang, 310022, China.

**Keywords:** esophageal squamous cell cancer, hsa_circ_0000700, hsa_miR-1229, cell proliferation, cell migration

## Abstract

Accumulating evidence has demonstrated that circular RNAs (circRNAs) are involved in the pathogenesis of cancer, including that of esophageal squamous cell carcinoma (ESCC). The current study aimed to investigate the role of hsa_circ_0000700 in ESCC. hsa_circ_0000700, miR-1229, and related functional gene expression was measured by RT-qPCR. To characterize the functions of hsa_circ_0000700 and miR-1229, ESCC cells were infected with hsa_circ_0000700-specific siRNA, miR-1229 mimics, and an inhibitor alone or in combination. Cell Counting Kit-8 (CCK8), colony formation, EdU, flow cytometry, and Transwell assays were employed to evaluate cell proliferation, apoptosis, and migration. Luciferase reporter and RNA immunoprecipitation assays were used to confirm the targeting relationship between hsa_circ_0000700 and miR-1229. Finally, a competing endogenous RNAs (ceRNA) network was built for hsa_circ_0000700, and miR-1229 targets were analyzed by bioinformatics. circ_0000700 expression was significantly upregulated in ESCC cell lines. Actinomycin D and RNase R treatment confirmed that circ_0000700 was more stable than its linear CDH9 mRNA form. Moreover, a cytoplasmic and nuclear fractionation assay suggested that circ_0000700 was mainly distributed in the cytoplasm of ECA-109 and TE-1 cells. *In vitro*, the proliferative and migratory capacities of ECA-109 and TE-1 cells were inhibited by knocking down circ_0000700 expression. Additionally, miR-1229 silencing reversed the circ_0000700-specific siRNA-induced attenuation of malignant phenotypes. Mechanistically, circ_0000700 was identified as a sponge of miR-1229 and could activate PRRG4, REEP5, and PSMB5 indirectly to promote ESCC progression. In summary, our results suggest that hsa_circ_0000700 functions as an oncogenic factor by sponging miR-1229 in ESCC.

## Introduction

Esophageal carcinoma (EC) is the 7^th^ most common malignant digestive tract cancer, ranking 6^th^ in mortality overall, with an estimated 572,000 new cases diagnosed and more than 509,000 deaths in 2018 worldwide, with the latter meaning that EC accounts for an estimated 1 in every 20 cancer-related deaths 1, 2. Histologically, most esophageal cancers are divided into esophageal adenocarcinoma (EAC) and esophageal squamous cell cancer (ESCC). ESCC is the most common histological type of EC 3. Among the EC cases, 90% are ESCC, which seriously threatens human health in developing countries 4. Currently, surgical treatment, chemotherapy, radiotherapy, and targeted molecular therapy for cancers are developing increasingly rapidly and can contribute to improving the outcomes of ESCC patients, but the 5-year survival rate of patients with ESCC is still less than 13% 4, 5. There are no obvious clinical features in the early stages of ESCC, resulting in the majority of ESCC patients having lost the opportunity for surgical treatment at the time of initial diagnosis. However, the exact diagnosis, prognostic prediction and underlying mechanism of esophageal cancer progression remain to be further elucidated. Therefore, there is an imperative need to identify novel sensitive biomarkers and therapeutic molecules to facilitate effective ESCC diagnosis and treatment.

Circular RNAs (circRNAs) are a particular type of endogenous noncoding RNA (ncRNA) characterized by a covalent closed-loop structure without a 5′ cap or 3′ tail and are more stable and free from degradation by RNA exonucleases than traditional linear RNAs 6, 7. In addition, it is noteworthy that the biological function of circRNAs is highly associated with specific tissues, cell types, and disease stages, making circRNAs promising noninvasive biomarkers for the diagnosis and prognosis of cancer patients. Many studies have demonstrated that circRNAs play a crucial role in the regulation of cellular biological events, including proliferation, apoptosis, and metastasis 5, 8. However, the biological functions and potential mechanisms of dysregulated circRNAs in the initiation and development of ESCC remain mostly unclear.

MicroRNAs (miRNAs) are endogenous, small ncRNA molecules that are 18-24 nucleotides in length and negatively regulate gene expression via base pairing with the 3′-untranslated region (3′-UTR) of target genes 9. Increasing evidence has demonstrated that circRNAs play an essential role as miRNA sponges via a competitive endogenous RNA (ceRNA) mechanism in many human cancers to attenuate the inhibitory effects of miRNAs on their target genes. For instance, Xu et al. showed that circ_0000654 facilitates ESCC cell proliferation, migration, invasion, and apoptosis *in vitro* by sponging miR-149-5p 10, and Huang et al. reported that the upregulation of circular RNA ciRS-7 expression enhances the migration and invasion of ESCC cells by affecting miR-7/KLF4 and NF-κB signaling 11. Likewise, the circRNA hsa_circRNA_100367 promotes KYSE-150R cell proliferation, migration, and radioresistance by binding to miR-217 and can serve as a promising biomarker and potential therapeutic target 12. These circRNA-miRNA signaling axes are related to the comprehensive malignant characteristics of ESCC, such as cancer cell apoptosis, metastasis, invasion, and radioresistance, which implies the potential value of circRNAs as novel therapeutic targets and biomarkers in ESCC.

With the development of high-throughput sequencing, although circRNAs were first discovered in 1976, a growing number of circRNAs have been confirmed to be linked to the progression of diverse malignancies, including ESCC. Similarly, Shi et al. observed 469 upregulated circRNAs and 275 downregulated circRNAs in ESCC by using the high-throughput Arraystar Human circRNA Array 13. In addition, Song et al. revealed that hsa_circ_0000337 (known as hsa_circRNA_100872), one of the top ten upregulated circRNAs, promotes cell proliferation, migration, and invasion in ESCC 7.

The novel circRNA hsa_circ_0000700 (gene symbol CHD9, also known as hsa_circRNA_001937), which was found to be remarkably upregulated with a fold change (FC)=6.517 and *P-value*=0.015 and is located at chr16:53155459-53155541, attracted our attention. Our study aimed to investigate the expression of hsa_circ_0000700; evaluate its roles in cell proliferation, apoptosis, and metastasis *in vitro*; and confirm that circ_0000700 functions as a ceRNA by sponging miR-1229 in ESCC cells. Our present study found that circ_0000700 expression was significantly upregulated in the ECA-109 and TE-1 cell lines. Additionally, we also demonstrated that siRNA knockdown of circ_0000700 expression and overexpression of miR-1229 inhibited the proliferation and migration of ECA-109 and TE-1 cells. Mechanistically, circ_0000700 was found to exacerbate ESCC progression via regulation of miR-1229/PRRG4/REEP5 and PSMB5 signaling. Therefore, circ_0000700 may provide a promising new avenue for the diagnosis and treatment of ESCC.

## Materials and Methods

### Cell culture

The esophageal cancer cell lines KYSE-410, KYSE-150, ECA-109, and TE-1 and the standard esophageal cell line HET-1A were obtained from the Cell Bank of the Chinese Academy of Sciences and incubated in RPMI-1640 medium (Gibco, Grand Island, NY, USA) supplemented with 10% fetal bovine serum (FBS; Gibco) and 1% penicillin/streptomycin (Invitrogen, CA, USA) at 37 ℃ in a 5% CO_2_ incubator.

### Quantitative reverse transcription-polymerase chain reaction (qRT-PCR)

Total RNA was extracted from KYSE-410, KYSE-150, ECA-109, TE-1, and HET-1A cells by using TRIzol Reagent (Invitrogen, CA, USA) according to the manufacturer's instructions. For circRNA analysis, cDNA was synthesized by using Prime Script^TM^ RT Master Mix (Takara, Japan), and for miRNA analysis, RNA was reverse transcribed into cDNA using the Mir-X miRNA First-Strand Synthesis Kit (Takara, Japan). The nucleus and cytoplasm were separated with NE-PER Nuclear and Cytoplasmic Extraction Reagents (Thermo Fisher Scientific) following the manufacturer's instructions. qRT-PCR was conducted with SYBR® Premix Ex Taq™ (Takara) on a CFX96 Touch Deep Well detection system (Bio-Rad, USA) with the following primers: circ_0000700, 5'-CTGGCTGAACATATCCCTTGGC-3' (forward) and 5'-TCCAAGGGATATGTTCAGCCA-3' (reverse); U6, 5'-CTCGCTTCGGCAGCACA-3' (forward) and 5'-AACGCTTCACGAATTTGCGT-3' (reverse); and GAPDH, 5'-AGA AGGCTGGGGCTCATTTG-3' (forward) and 5'-AGGGGCCATCCACAGTCTTC-3' (reverse). The entire sequence of the mature miR-1229 miRNA sequence was used as the miRNA-specific 5' primer, and the 3' primer for qRT-PCR was the mRQ 3' primer supplied with the kit. GAPDH was used as the reference gene for circ_0000700, and U6 was selected as the internal reference for miR-1229. The relative expression of the aforementioned targets was assessed by the 2^-ΔΔCt^ method.

### Actinomycin D and RNase R treatment

For actinomycin treatment, 2 mg/ml actinomycin D (Sigma, USA) was added to the cell culture medium. For RNase R treatment, total RNA (2 μg) was incubated for 15 min at 37 °C with or without 3 U/μg of RNase R (Epicentre Biotechnologies, China). After treatment with actinomycin D or RNase R, circ_0000700 expression and CHD9 mRNA expression levels were measured by qRT-PCR.

### Cell transfection

To inhibit the expression of circ_0000700 in the ESCC cell lines, three types of siRNA sequences and a negative control (NC) were purchased from GeneChem (Shanghai, China). The sequences were 5'-AACATATCCCTTGGATACTCT-3' for siCirc-1, 5'-ACATATCCCTTGGATACTCTA-3' for siCirc-2, 5'-CCCTTGGATACTCTAAGACCT-3' for siCirc-3, and 5'-AAUUCUCCGAAC GUGUCACGU-3' for the NC. For miR-1229 mimic and miR-1229 inhibitor transfection, miR-1229 mimics, the miR-1229 inhibitor, and the corresponding NC were synthesized by RiboBio (Guangzhou, China). The miRNA sequences were as follows: miR-1229 mimics, 5'-GUGGGUAGGGUUUGGGGGAGAGCG-3', and miR-1229 inhibitor, 5'-CGCUCUCCCCCAAACCCUACCCAC-3'. ECA-109 and TE-1 cells were transfected with Lipofectamine® 3000 (Thermo Fisher) according to the manufacturer's instructions, and the knockdown efficiency was measured by qRT-PCR.

### Cell viability and proliferation

Transfected cells were seeded in a 96-well plate at a cell density of 2 × 10^3^/ml per well. After culturing for 24, 48, 72, or 96 h, 10 μl of Cell Counting Kit-8 (CCK-8) proliferation solution (Beyotime Biotechnology, China) was added to each well and incubated for 2 h at 37 °C. The absorbance (OD value) at 450 nm was then analyzed using a microplate reader.

For the colony formation assay, ECA-109 and TE-1 cells were transfected with 50 nM si_circ_0000700 or related NC, and the transfected cells were plated in six-well plates at a density of 1000 cells per well and incubated in RPMI-1640 medium with 10% FBS at 37 °C. After 14 days, the visible colonies were fixed with 4% paraformaldehyde, stained with 0.2% crystal violet (Sigma), imaged, and then counted.

For the EdU assay, ECA-109 and TE-1 cells transfected with siCircRNA or the related NC were plated in 24-well plates at a density of 5 × 104 cells/well and incubated for another 48 h. Following the specified treatment, cells were then incubated in serum-free RPMI-1640 medium containing 10 μmol/L 5-ethynyl-2′-deoxyuridine (EdU, BeyoClick™ EdU-488, Beyotime Biotechnology, China) for 2 h. The cells were fixed with 4% paraformaldehyde and then underwent DAPI staining following the manufacturer's protocol. Images were captured using inverted fluorescence microscopy (Leica, Germany), and the green-colored positive cells were further quantified using ImageJ software (National Institutes of Health, Bethesda, MD, USA).

### Apoptosis analyses

Cell apoptosis was assessed on a FACSCanto II flow cytometer (BD Biosciences, San Jose, CA). Cells were transfected, collected and then treated with Annexin V-FITC (Beyotime, China) and propidium iodide (Keygen, China) at room temperature for 10 minutes each following the manufacturer's instructions.

### Cell migration

The migration of ECA-109 and TE-1 cells was evaluated with a Transwell assay carried out using Transwell chambers (Corning, NY). Briefly, 4×10^4^ transfected cells/well were suspended in serum-free RPMI 1640 medium and then added into the top compartment, and 500 μL of RPMI 1640 medium supplemented with 20% FBS was placed in the lower chamber. After incubation for 24 h, the cells on the lower filter surface were fixed, stained with 0.5% crystal violet for 15 minutes at room temperature, and digitally imaged in 6 random fields for each chamber using a microscope at 200× magnification.

### Western blot analysis

Transfected ECA-109 and TE-1 cells were lysed using precooled RIPA lysis buffer (Beyotime, China), and the protein concentration was quantified with the BCA Protein Assay Kit (Beyotime, China). Sodium dodecyl sulfate polyacrylamide gel electrophoresis (SDS-PAGE, 10%) was employed to separate the proteins, and then the proteins were transferred to polyvinylidene difluoride (PVDF) membranes (Millipore, Billerica, MA, USA). The membranes were blocked with 5% skim milk in a TBST solution for 1 h at room temperature and incubated with primary antibodies against Caspase 3 (1:500; ab13847, Abcam), Caspase 9 (1:2,000; ab32539, Abcam), Bax (1:1,000; ab32503, Abcam), Bcl-2 (1:2,000; ab182858, Abcam), E-cadherin (1:500; ab15148, Abcam), Vimentin (1:1,000; #12826, Cell Signaling Technology, Boston, USA), N-cadherin (1:1,000; #14215, Cell Signaling Technology, Boston, USA), and GAPDH (1:10,000; R1210-1, HuaBio, Hangzhou, China) at 4 °C overnight. Then, the membranes were incubated with an appropriate HRP-conjugated secondary antibody at room temperature for 2 h. Finally, the bands were visualized by enhanced chemiluminescence (Solarbio, China), and the intensity of the protein bands was analyzed using ImageJ software.

### Dual-luciferase reporter assays

Circular RNA Interactome (https://circinteractome.nia.nih.gov/index.html) 14 was used to predict the target miRNAs of circ_0000700. We then used a dual-luciferase reporter assay to confirm whether circ_0000700 contains miR-1229 binding sites. Full-length sequences of circ_0000700 with or without a mutant miR-1229 binding site were cloned into the luciferase vector pGL3 (Promega, Madison, USA). ECA-109 and TE-1 cells were cotransfected with the mutant or wild-type circ_0000700 luciferase vectors and miR-1229 mimics or the related NC with Lipofectamine® 3000 (Thermo Fisher). After 24 h of transfection, luciferase activity was estimated with a dual-luciferase reporter assay kit (RG027, Beyotime, China).

### RNA immunoprecipitation (RIP)

Experiments were performed based on the protocol of an EZMagna RIP kit (Merck, Darmstadt, Germany). In brief, ECA-109 and TE-1 cell lysates were prepared with RIP lysis buffer, and the supernatant was incubated with magnetic beads that were preconjugated with an anti-IgG or anti-Argonaute 2 (AGO2) antibody for 6 h at 4 °C. Subsequently, the magnetic beads were washed and incubated with protease K to digest the proteins. Finally, the levels of circ_0000700 and miRNA-1229 were detected by qRT-PCR.

### Bioinformatic analysis

TargetScan (http://www.targetscan.org/vert_72/) 15, miRPathDB 2.0 (https://mpd.bioinf.uni-sb.de/overview.html) 16, and miRTarBase (http://mirtarbase.mbc.nctu.edu.tw/php/index.php) 17 were employed to predict the target genes of miRNA-1229. In addition, the intersection of the aforementioned databases was analyzed through Venny 2.1 (https://bioinfogp.cnb.csic.Es/tools/venny/index.html). Then, a circRNA-miRNA-target regulatory network was visualized with Cytoscape software (v3.7.2, https://cytoscape.org/). To further investigate the biological function of the intersected targets, Kyoto Encyclopedia of Genes and Genomes (KEGG) pathway and Gene Ontology (GO) enrichment analyses were performed using the Metascape (http://metascape.org/gp/index.html#/main/step1) database 18. Additionally, overall survival (OS) related to the intersected targets was evaluated with Kaplan-Meier plotter (http://kmplot.com/analysis/index.php?p=service) 19. Finally, the mRNA expression levels of selected intersected targets were analyzed with the UALCAN cancer database (http://ualcan.path.uab.edu/analysis.html) 20.

### Statistical analysis

All data are expressed as the mean±standard deviation (SD), and statistical analyses were performed using SPSS 22.0 software (IBM, Chicago, USA). Statistical differences between two groups were evaluated by Student's t-test, and one-way analysis of variance (ANOVA) was used for comparison of three or more groups. *P<0.05* was considered statistically significant.

## Results

### Biological characteristics of hsa_circ_0000700 in ESCC cells

**Figure 1:**
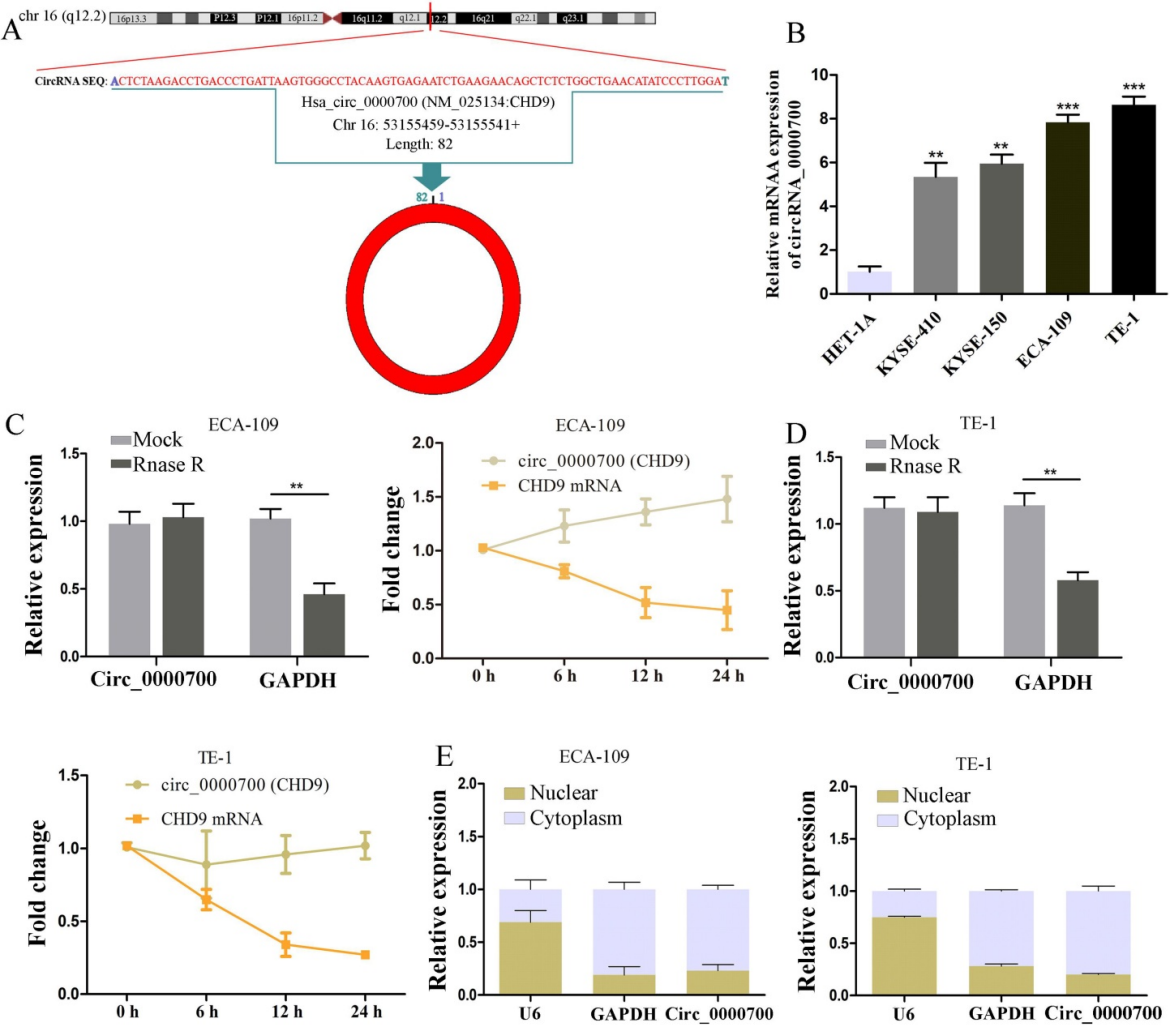
** Characterization of circ_0000700 in esophageal carcinoma cell lines.** (A) Schematic illustration showing the circularization of circ_0000700 by back splicing from chromosomal region 16q12.2. (B) The expression of circ_0000700 in esophageal carcinoma cell lines was detected by qRT-PCR. (C, D) The RNase R and actinomycin assays were used to evaluate the abundance of circ_0000700 and CHD9 mRNA in ECA-109 and TE-1 cells, respectively. (E) The expression levels of U6, GAPDH, circ_0000700 in cytoplasmic and nuclear fractions of ECA-109 and TE-1 cells were measured by qRT-PCR. ^*^P<0.05, ^**^P<0.01 and ^***^P<0.001.

According to the circBase database, hsa_circ_0000700 is generated from the q12.2 region of chromosome 16, with a length of 82 bases, and derived from CHD9 mRNA (Figure 1A). To explore the expression levels of circ_0000700 in ESCC cells, we used qRT-PCR to investigate circ_0000700 expression in a normal esophageal epithelial cell line (HET-1A) and ESCC cell lines (KYSE-410, KYSE-150, ECA-109, and TE-1), revealing that circ_0000700 expression in ESCC cells was remarkably increased (Figure 1B). We used ECA-109 and TE-1 cells as the experimental cell lines due to their high expression of circ_0000700. Additionally, RNase R exonuclease and actinomycin experiments were employed to confirm the circular nature of circ_0000700 in ECA-109 and TE-1 cells, respectively. The results suggested that circ_0000700 was resistant to the RNase R exonuclease and actinomycin, whereas linear CHD9 mRNA was not expressed in ECA-109 or TE-1 cells (Figure 1C and 1D). Moreover, a nuclear and cytoplasmic extraction experiment was conducted to investigate the subcellular localization of circ_0000700 in ECA-109 and TE-1 cells, and it was observed that most circ_0000700 was mainly concentrated in the cytoplasm of ECA-109 and TE-1 cells (Figure 1E). Taken together, these studies suggest that circ_0000700 is a stable circRNA that could be associated with the occurrence and development of ESCC and participate in posttranscriptional modification.

### Downregulation of circ_0000700 expression inhibits progression in ESCC cells

**Figure 2:**
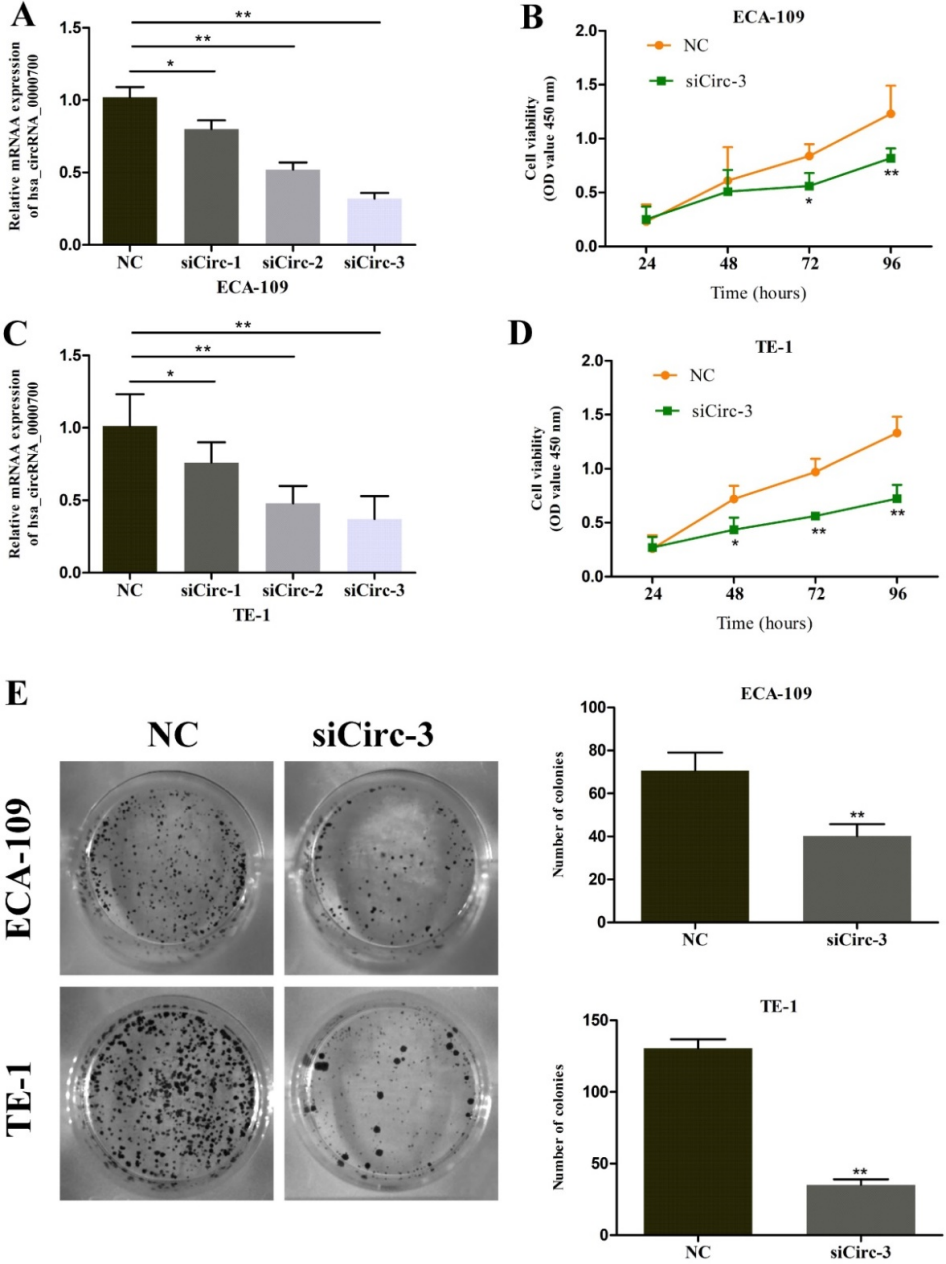
** Downregulation of circ_0000700 inhibits the proliferation of ESCC cells *in vitro*.** (A) The knockdown efficiency of circ_0000700 by siRNA interference in ECA-109 cells. (B) The effect of the silencing of circ_0000700 on cell viability of ECA-109 cells *in vitro* using the CCK-8 assay. (C) The knockdown efficiency of circ_0000700 by siRNA interference in TE-1 cells. (D) The effect of the silencing of circ_0000700 on cell viability of TE-1 cells *in vitro* using the CCK-8 assay. (E) Down-regulation of circ_0000700 inhibits the colony-forming ability of ECA-109 and TE-1 cells. ^*^P<0.05, ^**^P<0.01.

**Figure 3:**
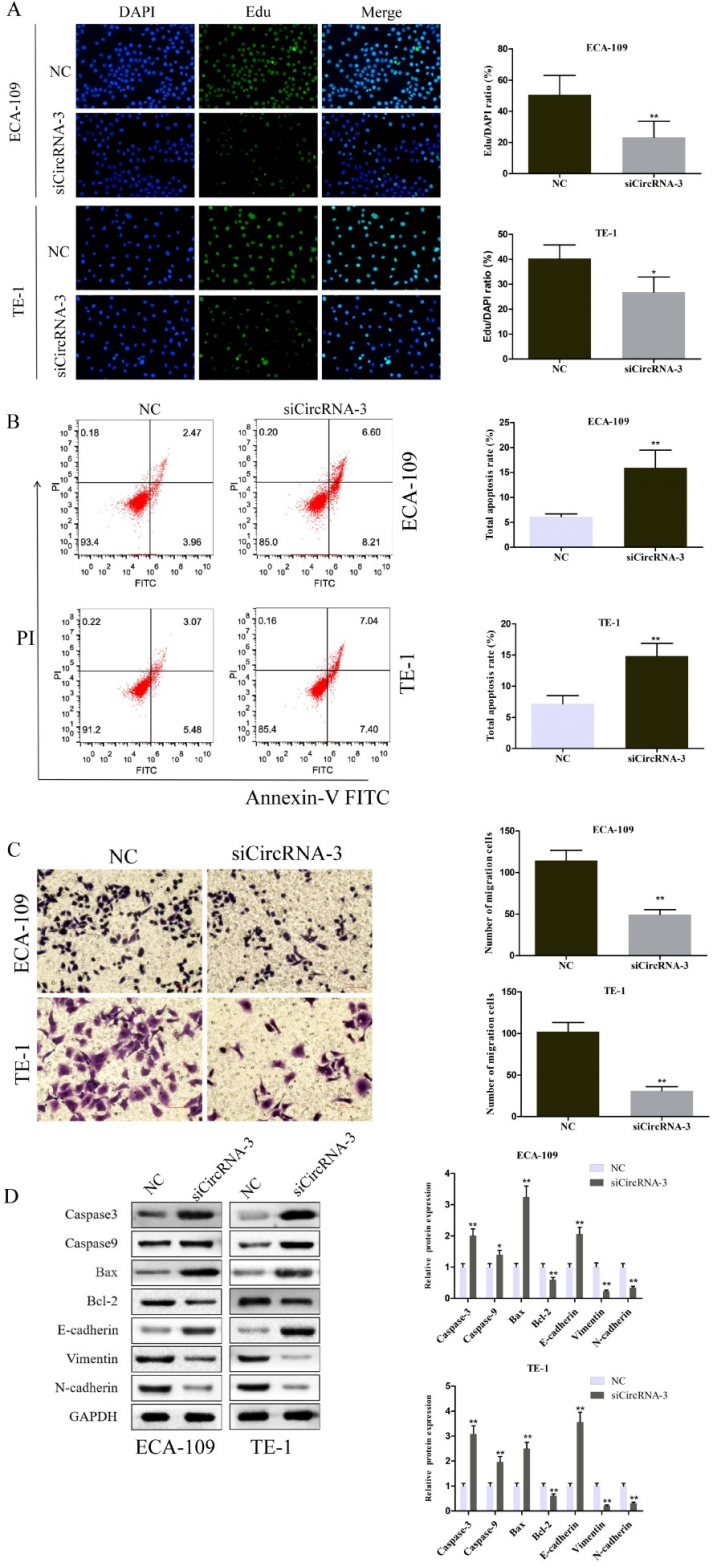
** Downregulation of circ_0000700 promotes apoptosis and suppresses proliferation, migration capacities of ESCC cells *in vitro*.** (A) EdU assay of ECA-109 and TE-1 cells transfected with negative control (NC) or circ_0000700 siRNA was performed to evaluate cell proliferation ability. (B) Flow cytometry apoptosis analysis of ECA-109 and TE-1 cells transfected with NC or circ_0000700 siRNA. (C) Cell migration assays were performed in ECA-109 and TE-1 cells using Transwell chambers. (D) Western blot showed that the silencing of circ_0000700 can promote the expression of Caspase-3, Caspase-9, Bax, and E-cadherin while restraining the protein levels of Bcl-2, vimentin, and N-cadherin. ^*^P<0.05, ^**^P<0.01.

To further evaluate the role of circ_0000700 in ESCC, circ_0000700 expression in ECA-109 cells was knocked down through transfection of siCirc-1/2/3. The knockdown efficiency was confirmed using qRT-PCR, and the results indicated that the siCirc-3 vector significantly decreased the endogenous circ_0000700 expression level (Figure 2A). Subsequently, a CCK-8 assay was employed to assess the effect of circ_0000700 on cell viability. The results showed that the viability of ECA-109 cells decreased observably after knocking down circ_0000700 expression (Figure 2B). With the depletion of circ_0000700, TE-1 cells displayed similar tendencies for knockdown efficiency and cell viability (Figure 2C and 2D). In addition, as shown in Figure 2E and 3A, compared with NC treatment, siRNA treatment of ECA-109 and TE-1 cells significantly decreased the number of cell colonies and EdU incorporation. Then, flow cytometric analyses were performed with ECA-109 and TE-1 cells. As illustrated in Figure 3B, cell apoptosis was apparently increased in the two cell lines after circ_0000700 silencing. As shown in Figure 3C, the number of migrated cells was significantly decreased in the circ_0000700-knockdown group compared with the NC group. In addition, by Western blot analysis of ESCC cells, compared with the NC group, the knockdown group showed downregulated circ_0000700 expression; significantly elevated Caspase-3, Caspase-9, Bax, and E-cadherin protein expression levels; and reduced Bcl-2, vimentin, and N-cadherin protein expression levels (Figure 3D). The above results indicated that circ_0000700 exerted a negative regulatory effect on ESCC cells.

### Circ_0000700 is a sponge of miR-1229

**Figure 4:**
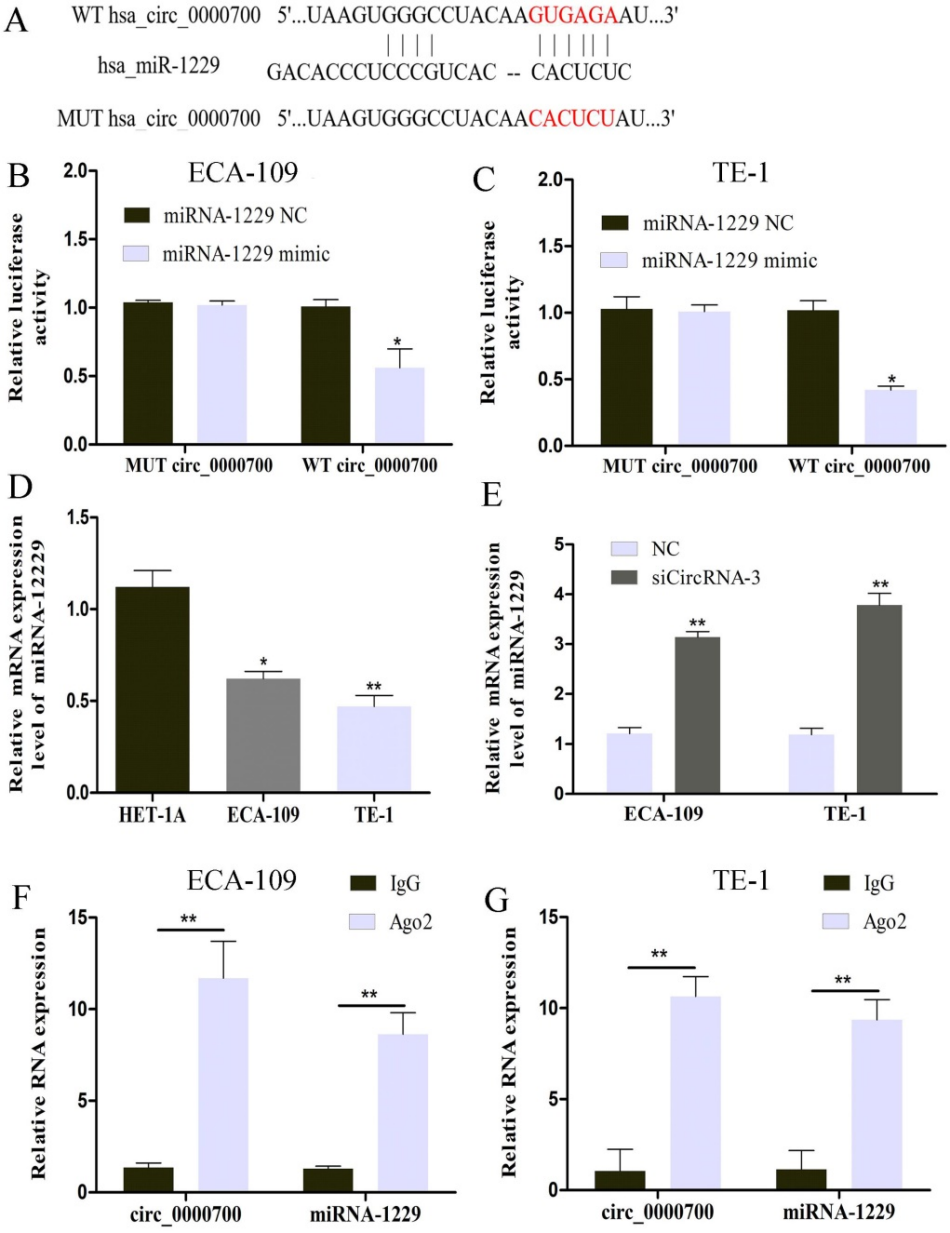
** circ_0000700 targets miR-1229 as a miRNA sponge.** (A) The possible binding sites of miR-1229 within circ_0000700. The luciferase activity decreased after cotransfection with miR-1229 mimics and wild-type circ_0000700 in ECA-109 (B) and TE-1 (C) cell lines. (D) The expression of miR-1229 in ECA-109 and TE-1 cells. (E) The miR-1229 level was increased in the ESCC ECA-109 and TE-1 cells transfected with NC or circ_0000700 siRNA. (F and G) RIP and qRT-PCR assays measure the differences of circ_0000700 and miR-1229 levels between the Ago2 and the IgG immunoprecipitation. ^*^P<0.05, ^**^P<0.01.

The cytoplasmic distribution of circ_0000700 indicated that it might function by sponging miRNAs. We then used the Circular RNA Interactome database to predict the potential miRNAs targeted by circ_0000700. As shown in Figure 4A, bioinformatic analysis results identified possible binding sites of circ_0000700 and miR-1229. To further investigate the relationship between circ_0000700 and miR-1229, a luciferase assay was conducted with ECA-109 and TE-1 cells, and the results showed that overexpression of miR-1229 led to tremendous attenuation of luciferase activity in the wild-type (WT) circ_0000700 group (Figure 4B and 4C). To explore the role of miR-1229 in ESCC, the miR-1229 expression level was also measured. As shown in Figure 4D, the expression level of miR-1229 was remarkably downregulated in ECA-109 and TE-1 cells in comparison to HET-1A cells. In addition, a qRT-PCR experiment also revealed that the miR-1229 expression level in ECA-109 and TE-1 cells was significantly increased through knockdown of circ_0000700 expression (Figure 4E). Subsequently, we also performed a RIP assay for Ago2 and found higher levels of circ_0000700 and miR-1229 in the Ago2 group than in the IgG group for ECA-109 and TE-1 cells (Figure 4F and 4G). Taken together, these findings indicated that miR-1229 might be a significant downstream target of circ_0000700 in ESCC.

### miR-1229 inhibited ESCC cell proliferation and migration

**Figure 5:**
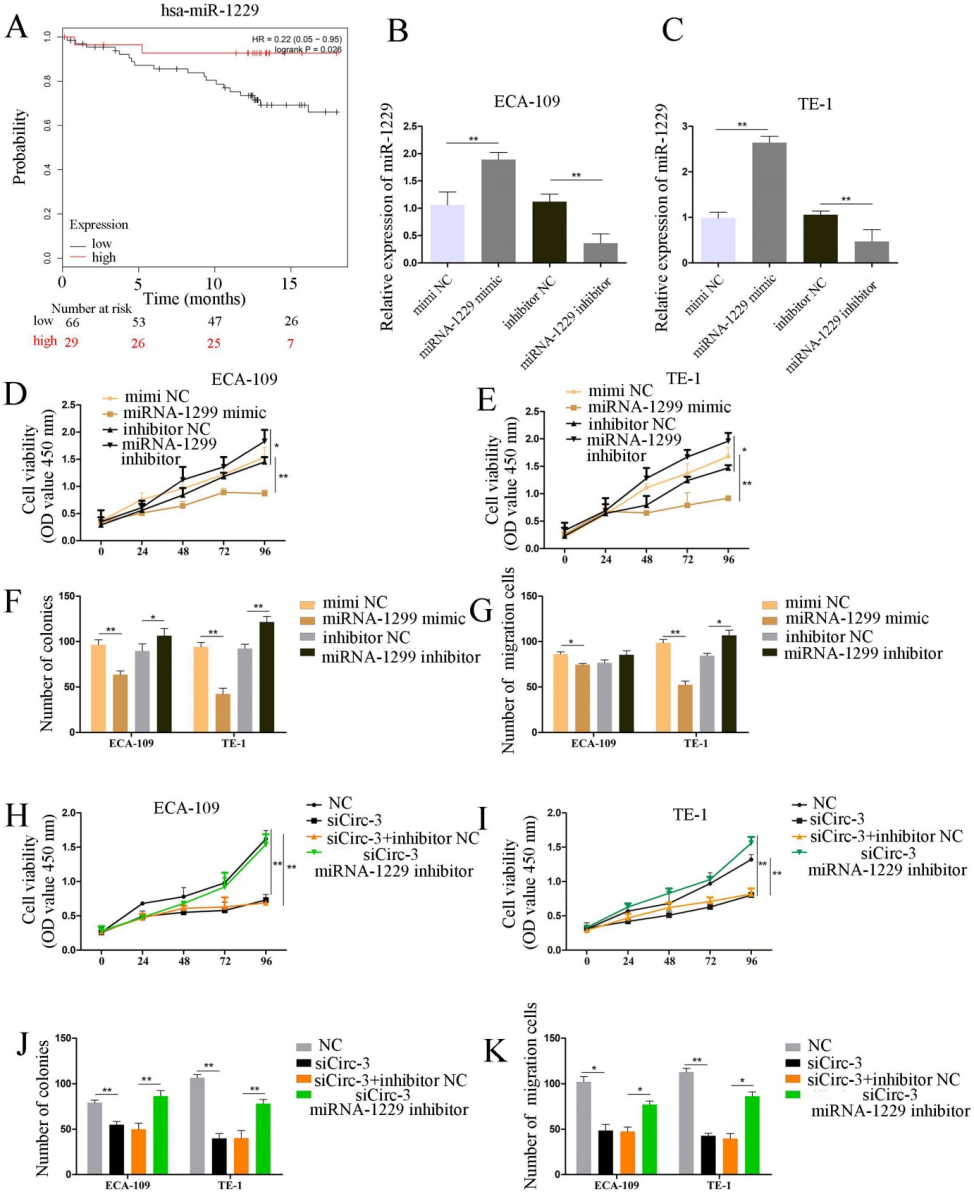
** miR-1229 acts as a tumor suppressor gene in ECA-109 and TE-1 cells, and the silencing of miR-1229 effectively reverses circ_0000700 siRNA induced inhibition of proliferation and migration.** (A) The overall survival curve of ESCC patients with low and high miR-1229 expression. (B and C) The expression level of miR-1229 in ECA-109 and TE-1 cells transfected with miR-1229 mimics and miR-1229 inhibitor. (D, E, F, and G) Cell proliferation, colony formation, and cell migration assays were determined in ECA-109 and TE-1 cells following transfection with miR-1229 inhibitor, miR-1229-NC or miR-1229 mimics. (H, I, J, and K) ECA-109 and TE-1 cells transfected with circ_0000700-NC, miR-1229 inhibitor NC, miR-1229 inhibitor, or circ_0000700 siRNA alone or simultaneously. Then, the cell proliferation and migration were, respectively, assessed by CCK-8 (H and I), clone assay (J), and the transwell migration (K) assay. ^*^P<0.05, ^**^P<0.01.

To investigate the clinical significance of miR-1229 in ESCC, we divided ESCC patients into high and low expression groups by using Kaplan-Meier plotter (http://kmplot.com/). As demonstrated in Figure 5A, a low miR-1229 expression level was associated with unfavorable overall survival (OS, hazard ratio [HR] =0.22; 95% CI: 0.05-0.95, *P*=0.026). Accordingly, to elucidate the biological effect of miR-1229 in ESCC, miR-1229 mimics or a miR-1229 inhibitor was transfected into ECA-109 and TE-1 cells, and then qRT-PCR analysis was conducted to evaluate the transfection efficiency, suggesting that we successfully overexpressed and knocked down miR-1229, respectively, in the two cell lines (Figure 5B and 5C). Compared with the NC group, the miR-1229 mimic group showed reduced cell viability, colony numbers and cell migration (Figure 5D-5G). Furthermore, inhibition of miR-1229 expression had the opposite effects (Figure 5D-5G). To further validate the results for the circ_0000700/miR-1229 axis, we performed rescue experiments *in vitro*. As shown in Figure 5H-5K, the miR-1229 inhibitor partly reversed the effects of circ_0000700-specific siRNA on the proliferation and migration of ESCC cells. In summary, these data reveal that the circ_0000700/miR-1229 axis is involved in ESCC progression.

### Bioinformatic analysis of candidate targets of miR-1229

**Figure 6:**
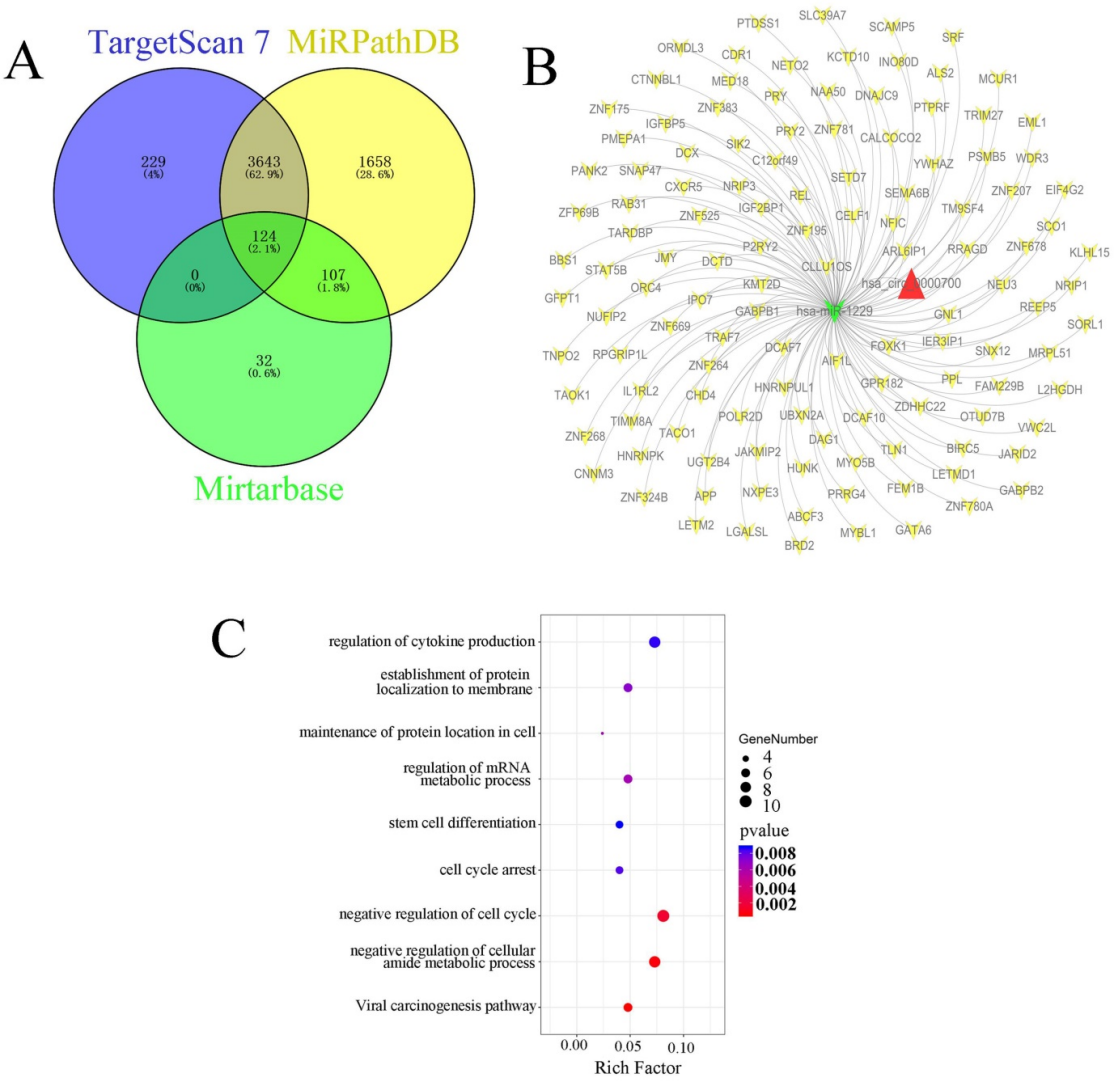
** Identification and functional enrichment analysis of predicted targets of miR-1229** (A) Venn diagram of predicted target genes from the intersection of TargetScan, miRPathDB 2.0, and miRTarBase database. (B) The ceRNA network of circ_0000700-miR-1229-mRNA. The red triangle indicates circ_0000700, green “V” indicates miR-1229, and yellow “V” indicates the mRNAs. (C) The KEGG pathway and GO enrichment analysis of predicted target genes.

**Figure 7:**
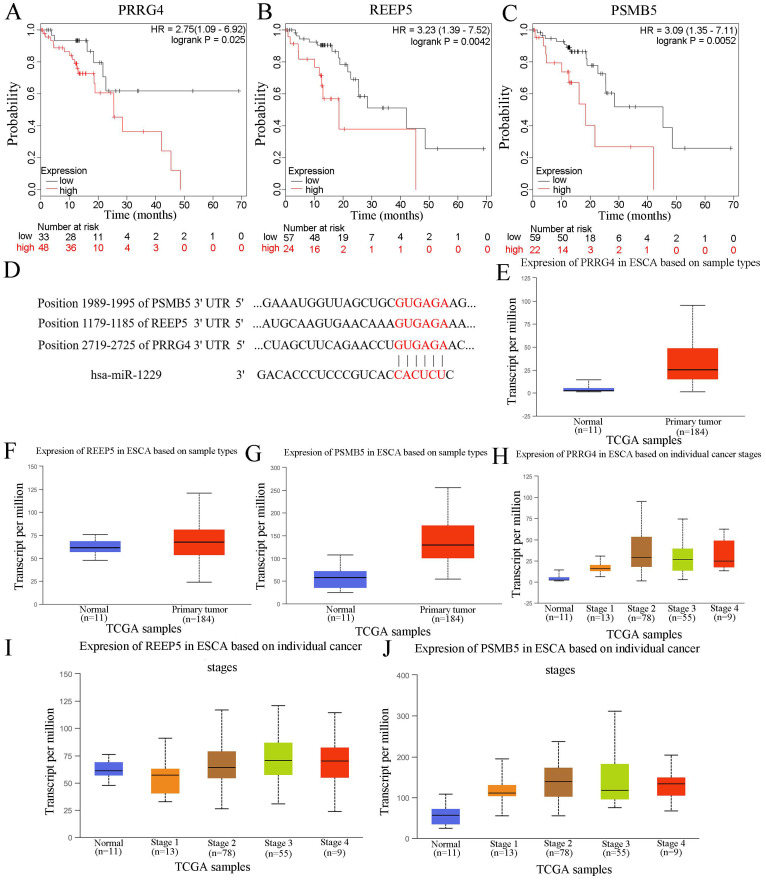
** The relationship of PRRG4, REEP5, and PSMB5 with clinical parameters and survival of ESCC patients.** (A, B, C) The prognostic value of PRRG4, REEP5, and PSMB5 in ESCC patients according to the Kaplan Meier plotter. (D) Binding sites of the miRNA hsa_miR_1299 in the target genes, PRRG4, REEP5, and PSMB5. (E, F, G) The relative expression of PRRG4, REEP5, and PSMB5 in primary tumor tissues compared with normal tissues based on the UALCAN database. (H, I, J) The relative expression of PRRG4, REEP5, and PSMB5 was correlated with tumor stages in ESCC.

In this study, we also predicted the candidate target genes of miR-1229 with the TargetScan, miRPathDB, and miRTarBase databases (Figure 6A). Next, we intersected the results from the aforementioned databases and found that 124 predicted target genes, including DCTD, ALS2, REEP5, NXPE3, CHD4, and PSMB5, could be directly targets of miR-1229. Then, the hsa_circ_0000700/hsa_miR_1229/mRNA axis interaction network was constructed (Figure 6B). To determine the biological significance of target genes, KEGG pathway and GO enrichment analyses were performed. We observed that the target genes were significantly associated with the viral carcinogenesis pathway, regulation of the cellular amide metabolic process, negative regulation of the cell cycle, and regulation of cytokine production (Figure 6C). These results suggested that the predicted genes exhibited a close relationship with the development and progression of ESCC. Moreover, the clinical significance of predicted target gene expression in ESCC was further assessed according to Kaplan-Meier survival analysis. We found that low expression of each of 26 target genes including CELF1, CHD4, DCAF10, DNAJC9, FOXK1, GATA6, GNL1, GPR182, HNRNPK, HNRNPUL1, IGF2BP1, JMY, L2HGDH, NEU3, NRIP3, ORMDL3, OTUD7B, POLR2D, SCO1, SIK2, SLC39A7, TIMM8A, TNPO2, UBXN2A, WDR3, and ZFP69B was significantly associated with poor OS (Figure S1). In addition, Kaplan-Meier analysis also revealed that high expression of PRRG4 (HR=2.75; 95% CI: 1.09-6.92, *P*=0.025), REEP5 (HR=3.23; 95% CI: 1.39-7.52, *P*=0.0042), and PSMB5 (HR=3.09; 95% CI: 1.35-7.11, *P*=0.0052) was associated with lower survival rates (Figure 7A-7C). In addition, the predicted binding sites of hsa_miR_1229 within PRRG4, REEP5, and PSMB5 are shown in Figure 7D. Finally, we found that the expression of PRRG4, REEP5, and PSMB5 in primary tumor tissues was significantly higher than that in normal tissues (Figure 7E-7G). Moreover, as shown in Figure 7H-7J, the expression levels of PRRG4, REEP5, and PSMB5 were significantly associated with TNM stage to a certain extent. Therefore, we speculated that downregulation of miR-1229 expression promoted proliferation and migration in ESCC by targeting PRRG4, REEP5, and PSMB5.

## Discussion

Esophageal squamous cell cancer (ESCC) is a malignant tumor with high morbidity and mortality, and clinical outcomes are not satisfactory due to the lack of early diagnosis and limitations of conventional treatment strategies. Therefore, it is of clinical significance to further investigate novel therapeutic targets and sensitive diagnostic biomarkers in ESCC to improve the poor patient prognosis. Increasing evidence indicates that circRNAs, characterized by covalently closed-loop structures, are closely associated with the occurrence and development of multiple cancers, including breast cancer 21, gastric cancer 22, and ESCC 23, making them potential therapeutic targets.

In this study, a novel differentially expressed circRNA named hsa_circ_0000700 was identified based on the research of Shi et al 13, and the expression of this circRNA was remarkably upregulated in ESCC cell lines compared with HET-1A cells. Subsequently, the biological characteristics of circ_0000700 were evaluated in ECA-109 and TE-1 cells, and the results showed that circ_0000700 was reasonably static and highly expressed in the cytoplasm of ESCC cells, which indicates that the underlying molecular mechanism of circ_0000700 is possible posttranscriptional regulation. The above results suggested that circ_0000700 might play an essential role in the biological events of ESCC. Next, we knocked down the expression of circ_0000700 in ECA-109 and TE-1 cells and assessed the effects on cell viability, proliferation, apoptosis, and migration. Functional experiments confirmed that knocking down circ_0000700 expression significantly inhibited the proliferation and migration of ESCC cells, suggesting that circ_0000700 serves as an oncogene in ESCC progression.

Numerous studies have shown that circRNAs may serve as oncogenic or tumor-suppressive genes by regulating epithelial-mesenchymal transition (EMT). For example, hsa_circ_0006948 can induce EMT to promote migration and invasion by sponging miR-490-3p in ESCC 24. He et al. found that overexpression of circVRK1 depressed cell proliferation, migration, and EMT and reversed radioresistance through activation of PTEN mediated by sponging miR-624-3p and inhibiting the PI3K/AKT signaling pathway in ESCC progression 25. Moreover, we found that circ_0000700 not only promoted apoptosis in ESCC cell lines *in vitro* but also had a regulatory role in EMT in ESCC cells. However, the potential mechanism by which circ_0000700 participates in the EMT process needs further experimental verification.

Currently, it has been reported that circRNAs can act as miRNA sponges to participate in posttranscriptional regulation in multiple types of tumors 26. For instance, the circRNA SMARCA5 is able to suppress the proliferation of cervical cancer cells by modulating miR-432 27. circVAPA was found to promote migration and invasion in breast cancer by binding miR-130a-5p in the cytoplasm 28. Overexpressed circLARP4 inhibits cell proliferation and migration but promotes cell apoptosis by sponging miR-1323, which positively regulates PTEN expression in ESCC 29. Using bioinformatic analysis, we predicted miR-1229 as a potential target of circ_0000700 and further confirmed this result in dual-luciferase reporter and RIP assays, as well as functional enrichment analysis. Our data suggested that miR-1229 overexpression significantly inhibited ECA-109A and TE-1 cell proliferation and migration and that miR-1229 deficiency in circ_0000700-deficient cells substantially abolished circ_0000700-circKDM4C-mediated biological effects. Previously, miR-1229 was reported to participate in the viability and apoptosis of FLT3-ITD-positive acute myeloid leukemia cells 30. In renal cell carcinoma, circ-EGLN3 knockdown suppresses proliferation and aggressiveness by targeting miR-1229 31. Additionally, Meng L et al. reported that miR-1229 promotes starvation- or rapamycin-induced autophagy in ESCC cells by directly binding to epidermal growth factor receptor (EGFR) 32. In addition, the GO and KEGG enrichment analyses of targets of miR-1229 suggested that miR-1229 might affect the viral carcinogenesis pathway and regulation of the cell cycle. According to the above results, circ_0000700 knockdown could decrease the degradation of miR-1229 to restrain the growth of ESCC.

There are currently many papers suggesting that the circRNA-miRNA-mRNA axis is related to cancer progression. In this study, we predicted candidate genes targets of miR-1229, among which PRRG4, REEP5, and PSMB5 were associated with a poor prognosis and highly expressed in tumor tissues. The PRRG4 gene can help in the autistic symptoms of WAGR (Wilm's tumor, Aniridia, genitourinary malformations, and mental retardation) syndrome 33. In addition, Liu et al. identified proline rich and Gla domain 4 (PRRG4) as a potential prognostic marker in cholangiocarcinoma (CCA) 34. Further study revealed that depletion of receptor expressing enhancing protein 5 (REEP5) significantly reduced growth and invasion in lung cancer 35. Studies have shown that proteasome subunit beta 5 (PSMB5) is overexpressed in triple-negative breast cancer and significantly associated with a poor prognosis 36 and that knockdown of PSMB5 gene expression inhibits MDA-MB-231 cell growth and migration 37. These results also suggested that PRRG4, REEP5, and PSMB5 might serve as novel cancer therapeutic targets. Hence, we hypothesized that circ_0000700 may promote the proliferation and migration of ESCC cells by increasing PRRG4, REEP5, and PSMB5 expression. However, in the current study, the specific relationships between miR-1229 and the candidate targets still requires confirmation with a luciferase assay. Therefore, the present study had certain limitations. First, the expression levels and prognostic significance of circ_0000700 and miR-1229 should be evaluated based on clinical samples. Moreover, further functional experiments investigating the relationships among circ_0000700, miR-1229, PRRG4, REEP5, and PSMB5 are suggested in the future to confirm the role of circ_0000700 in this mechanism.

In summary, the present study revealed that hsa_circ_0000700 expression was upregulated in ESCC cells and that silencing hsa_circ_0000700 contributed to restraining the proliferation and migration of ECA-109A and TE-1 cells through binding to miR-1229. Our results suggest that the hsa_circ_0000700/miR-1229/PRRG4-REEP5-PSMB5 axis could be a promising therapeutic target in ESCC.

## Supplementary Material

Supplementary figure.

## Abbreviations

CircRNAs: circular RNAs
ESCC: esophageal squamous cell carcinoma
CCK8: Cell Counting Kit-8
EC: esophageal carcinoma
EAC: esophageal adenocarcinoma
NcRNAs: noncoding RNAs
miRNAs: MicroRNAs
3′-UTRs: 3′-untranslated regions
ceRNA: competitive endogenous RNA
qRT-PCR: quantitative reverse transcription-polymerase chain reaction
NC: negative control
SDS-PAGE: sodium dodecyl sulfate polyacrylamide gel electrophoresis
PVDF: polyvinylidene difluoride
RIP: RNA immunoprecipitation
AGO2: argonaute 2
KEGG: Kyoto Encyclopedia of Genes and Genomes
GO: Gene Ontology
OS: overall survival
SD: standard deviation
WT: wild-type
PRRG4: proline rich and Gla domain 4
CCA: cholangiocarcinoma
REEP5: receptor expressing enhancing protein 5
PSMB5: proteasome subunit beta 5
