# Supplementary material for: Circular RNA hsa_circ_0000700 promotes cell proliferation and migration in Esophageal Squamous Cell Carcinoma by sponging miR-1229
Source: J Cancer. 2021 Mar 5;12(9):2610–23. doi: 10.7150/jca.47112 (PMC8040728; doi:10.7150/jca.47112)
Supplement: Supplementary file 1 — Supplementary figure. [file jcav12p2610s1.pdf]

Supplementary file Figure S1

Figure S1 The overall survival curve of 26 target genes based on the Kaplan Meier plotter

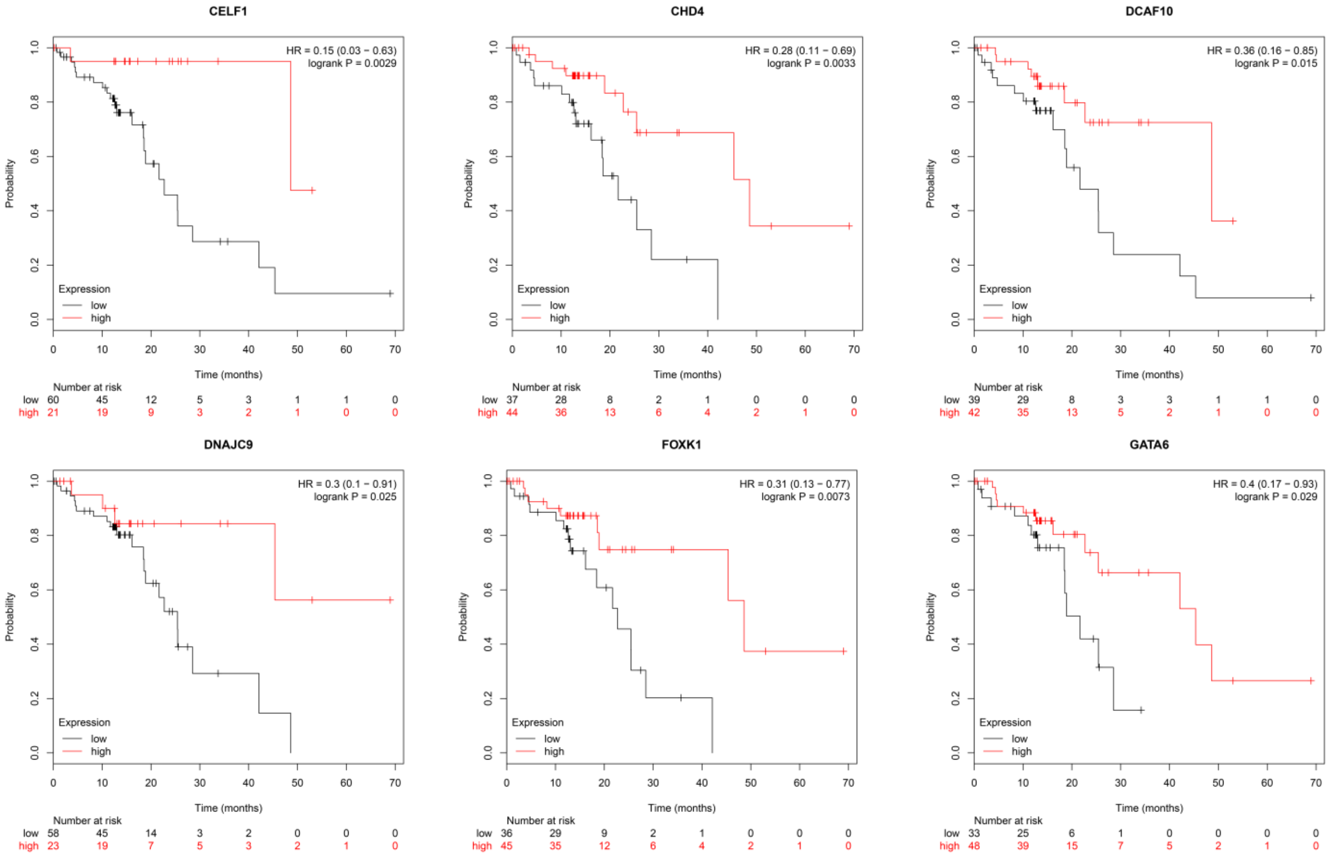

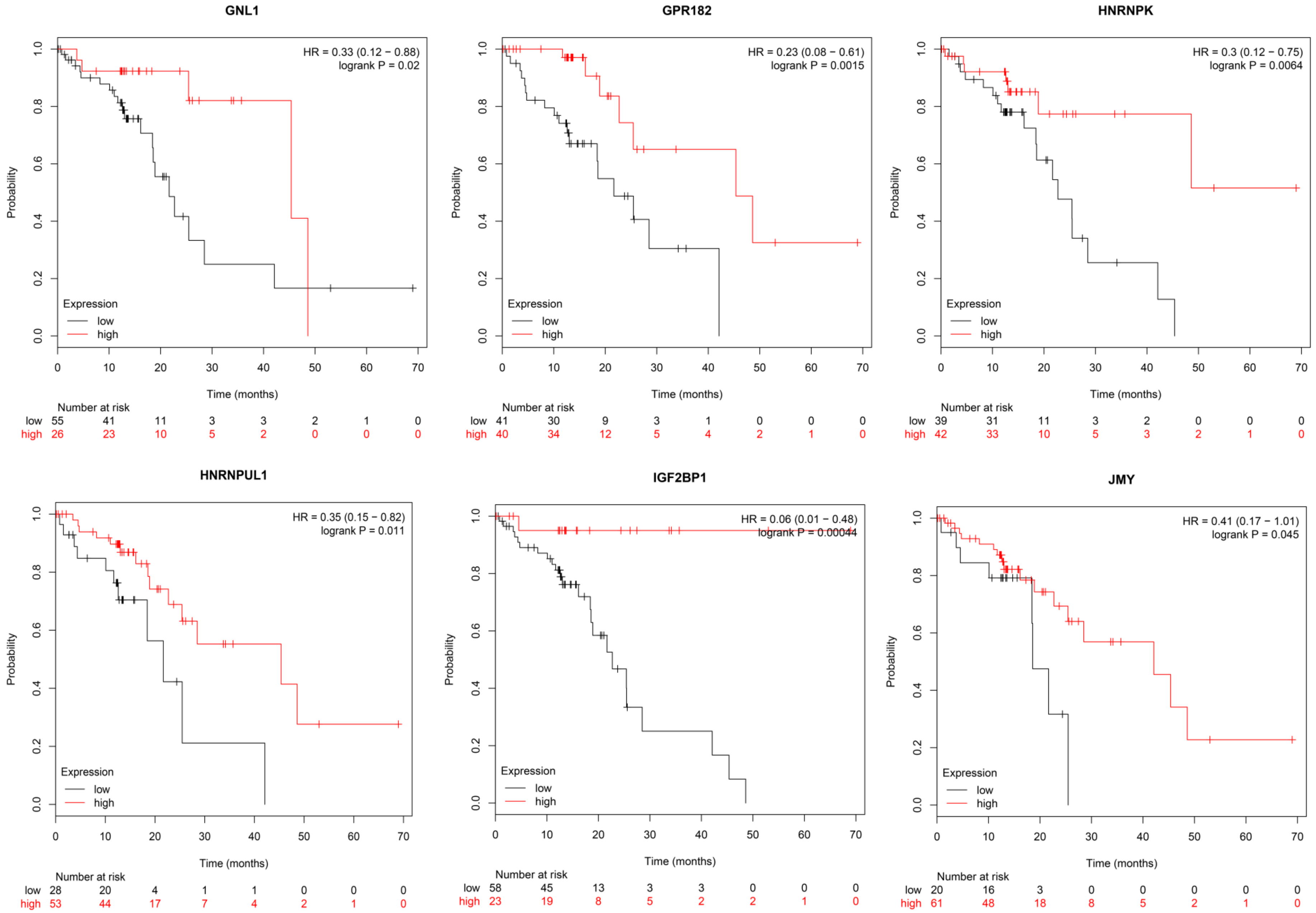
GNL1
HR = 0.33 (0.12 − 0.88)
logrank P = 0.02
Expression
low
high
Probability
Time (months)
Number at risk
low 55 41 11 3 3 2 1 0
high 26 23 10 5 2 0 0 0
GPR182
HR = 0.23 (0.08 − 0.61)
logrank P = 0.0015
Expression
low
high
Probability
Time (months)
Number at risk
low 41 30 9 3 1 0 0 0
high 40 34 12 5 4 2 1 0
HNRNPK
HR = 0.3 (0.12 − 0.75)
logrank P = 0.0064
Expression
low
high
Probability
Time (months)
Number at risk
low 39 31 11 3 2 0 0 0
high 42 33 10 5 3 2 1 0
HNRNPUL1
HR = 0.35 (0.15 − 0.82)
logrank P = 0.011
Expression
low
high
Probability
Time (months)
Number at risk
low 28 20 4 1 1 0 0 0
high 53 44 17 7 4 2 1 0
IGF2BP1
HR = 0.06 (0.01 − 0.48)
logrank P = 0.00044
Expression
low
high
Probability
Time (months)
Number at risk
low 58 45 13 3 3 0 0 0
high 23 19 8 5 2 2 1 0
JMY
HR = 0.41 (0.17 − 1.01)
logrank P = 0.045
Expression
low
high
Probability
Time (months)
Number at risk
low 20 16 3 0 0 0 0 0
high 61 48 18 8 5 2 1 0

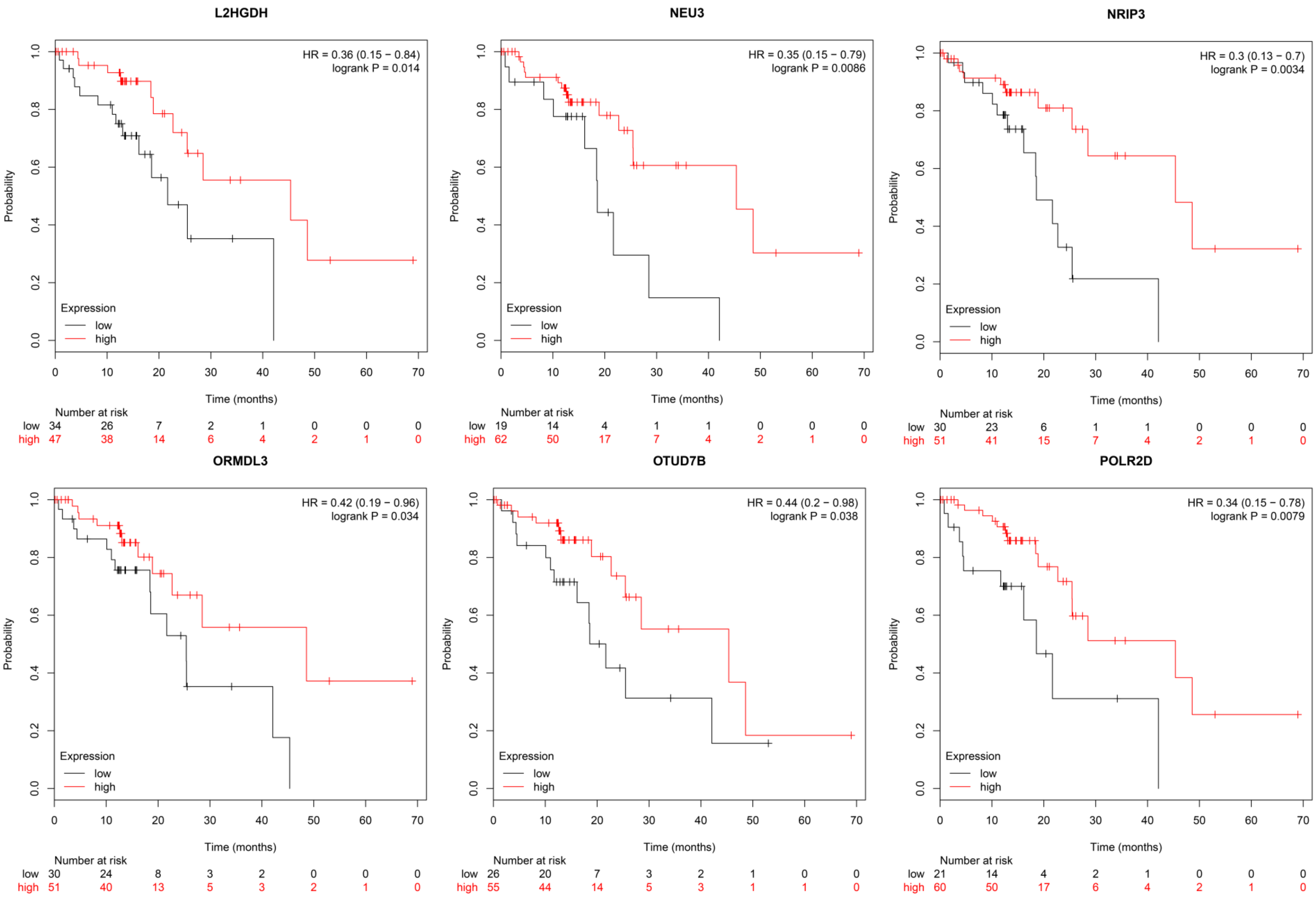
L2HGDH
HR = 0.36 (0.15 − 0.84)
logrank P = 0.014
Expression
low
high
Probability
Time (months)
Number at risk
low 34 26 7 2 1 0 0 0
high 47 38 14 6 4 2 1 0
NEU3
HR = 0.35 (0.15 − 0.79)
logrank P = 0.0086
Expression
low
high
Probability
Time (months)
Number at risk
low 19 14 4 1 1 0 0 0
high 62 50 17 7 4 2 1 0
NRIP3
HR = 0.3 (0.13 − 0.7)
logrank P = 0.0034
Expression
low
high
Probability
Time (months)
Number at risk
low 30 23 6 1 1 0 0 0
high 51 41 15 7 4 2 1 0
ORMDL3
HR = 0.42 (0.19 − 0.96)
logrank P = 0.034
Expression
low
high
Probability
Time (months)
Number at risk
low 30 24 8 3 2 0 0 0
high 51 40 13 5 3 2 1 0
OTUD7B
HR = 0.44 (0.2 − 0.98)
logrank P = 0.038
Expression
low
high
Probability
Time (months)
Number at risk
low 26 20 7 3 2 1 0 0
high 55 44 14 5 3 1 1 0
POLR2D
HR = 0.34 (0.15 − 0.78)
logrank P = 0.0079
Expression
low
high
Probability
Time (months)
Number at risk
low 21 14 4 2 1 0 0 0
high 60 50 17 6 4 2 1 0

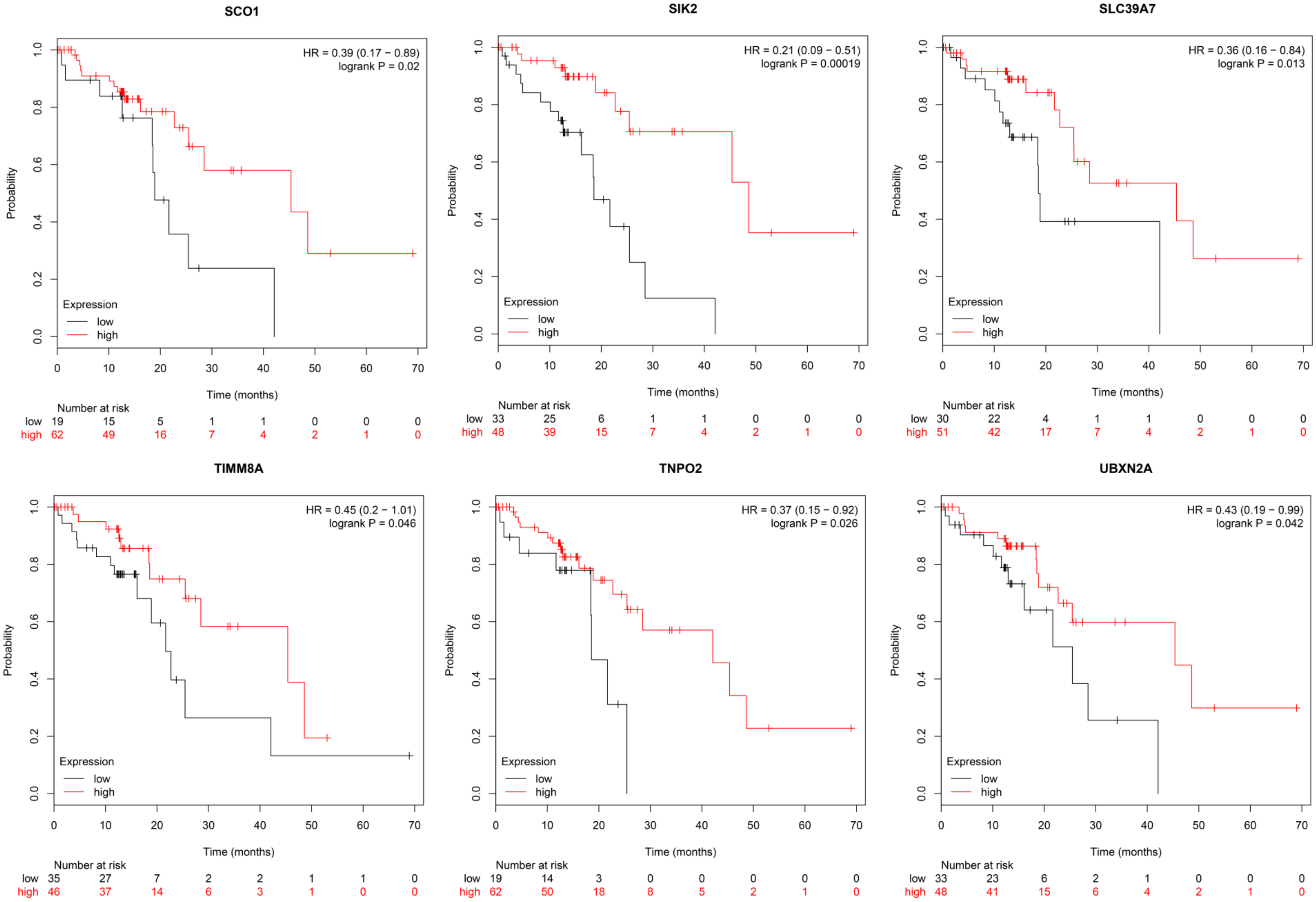
SCO1
HR = 0.39 (0.17 − 0.89)
logrank P = 0.02
Probability
Time (months)
Expression
low
high
Number at risk
low 19 15 5 1 1 0 0 0
high 62 49 16 7 4 2 1 0
SIK2
HR = 0.21 (0.09 − 0.51)
logrank P = 0.00019
Probability
Time (months)
Expression
low
high
Number at risk
low 33 25 6 1 1 0 0 0
high 48 39 15 7 4 2 1 0
SLC39A7
HR = 0.36 (0.16 − 0.84)
logrank P = 0.013
Probability
Time (months)
Expression
low
high
Number at risk
low 30 22 4 1 1 0 0 0
high 51 42 17 7 4 2 1 0
TIMM8A
HR = 0.45 (0.2 − 1.01)
logrank P = 0.046
Probability
Time (months)
Expression
low
high
Number at risk
low 35 27 7 2 2 1 1 0
high 46 37 14 6 3 1 0 0
TNPO2
HR = 0.37 (0.15 − 0.92)
logrank P = 0.026
Probability
Time (months)
Expression
low
high
Number at risk
low 19 14 3 0 0 0 0 0
high 62 50 18 8 5 2 1 0
UBXN2A
HR = 0.43 (0.19 − 0.99)
logrank P = 0.042
Probability
Time (months)
Expression
low
high
Number at risk
low 33 23 6 2 1 0 0 0
high 48 41 15 6 4 2 1 0

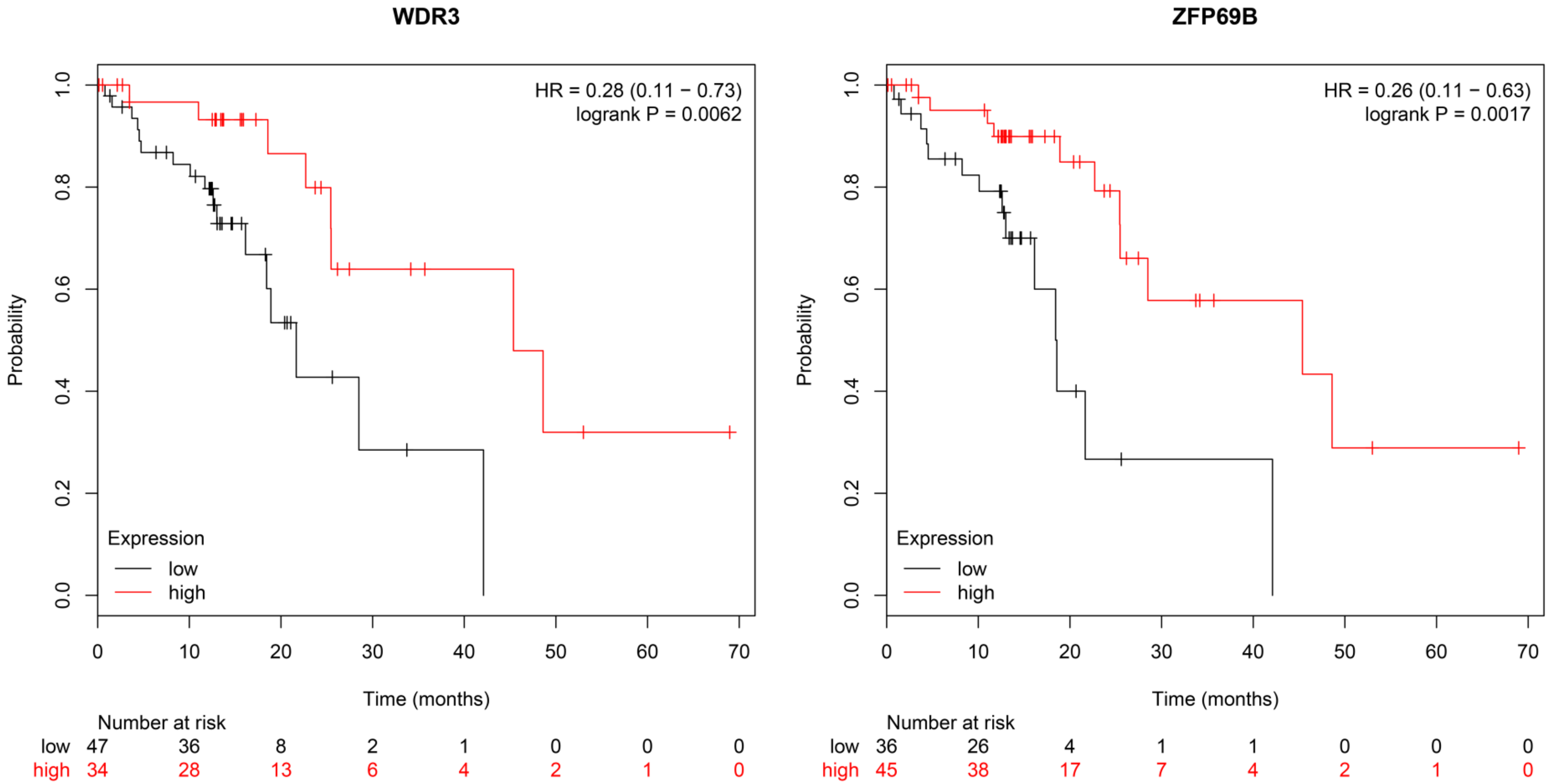
WDR3
HR = 0.28 (0.11 − 0.73)
logrank P = 0.0062
Probability
Expression
low
high
Time (months)
Number at risk
low 47 36 8 2 1 0 0 0
high 34 28 13 6 4 2 1 0
ZFP69B
HR = 0.26 (0.11 − 0.63)
logrank P = 0.0017
Probability
Expression
low
high
Time (months)
Number at risk
low 36 26 4 1 1 0 0 0
high 45 38 17 7 4 2 1 0
